# Prevalence and Correlates of Probable Nonalcoholic Steatohepatitis (NASH) in Pars Cohort Study

**DOI:** 10.34172/aim.30020

**Published:** 2024-11-01

**Authors:** Amin Nakhostin-Ansari, Iman Menbari Oskouie, Reyhaneh Aghajani, Mohammad Mehdi Khadembashiri, Mohammad Ahmadi, Abdollah Gandomkar, Fatemeh Malekzadeh, Hossein Poustchi, Mohammad Reza Fattahi, Amir Anushiravani, Reza Malekzadeh

**Affiliations:** ^1^Neuromusculoskeletal Research Center, Iran University of Medical Sciences, Tehran, Iran; ^2^Students’ Scientific Research Center, Tehran University of Medical Sciences, Tehran, Iran; ^3^Non-Communicable Disease Research Center, Shiraz University of medical Sciences, Shiraz, Iran; ^4^Liver and Pancreatobiliary Diseases Research Center, Digestive Disease Research Institute, Shariati Hospital, Tehran University of Medical Sciences, Teheran, Iran; ^5^Gastroenterohepatology Research Center, Shiraz University of Medical Sciences, Shiraz, Iran

**Keywords:** Epidemiology, Iran, Nonalcoholic steatohepatitis, Prevalence

## Abstract

**Background:**

Studies on the prevalence of nonalcoholic steatohepatitis (NASH) and the factors associated with its high prevalence among Iranian people are limited. This study evaluated the prevalence of NASH and its associated factors among Iranian adults using Pars Cohort Study (PCS) data.

**Methods:**

This cross-sectional study was conducted based on PCS, which includes 40-75-year-old adults from the Valashahr area. NASH was defined as alanine aminotransferase (ALT) higher than 40 U/L without evidence of hepatitis B or C infections. The prevalence of NASH and its associations with basic and demographic characteristics, socioeconomic characteristics, medical history, gastrointestinal symptoms, and laboratory tests were evaluated.

**Results:**

Overall, 8734 patients, including 3917 men (44.8%), were enrolled in this study. The mean age of participants was 52.62 years (SD=9.68), and 605 individuals had NASH (6.9%). In the regression analysis, in contrast to female gender (OR=0.31, 95% CI=0.249‒0.386, *P*<0.001) and age (OR=0.951, 95% CI=0.941‒0.962, *P*<0.001), history of heart disease (OR=1.499, 95% CI=1.146‒1.962, *P*=0.003), history of diabetes (OR=1.523, 95% CI=1.162‒1.995, *P*=0.002), hypertension (OR=1.241, 95% CI=1.023‒1.506, *P*=0.029), being overweight or obese (OR=2.192, 95% CI=1.755‒2.737, *P*<0.001), being in the richest or second richest wealth index quantiles (OR=1.315, 95% CI=1.107‒1.156, *P*=0.002), and increased waist circumference (OR=1.409, 95% CI=1.107‒1.793, *P*<0.005) were independently associated with a higher risk of having NASH.

**Conclusion:**

In this study, we determined the prevalence of NASH and found male gender, younger age, history of heart disease, history of diabetes, hypertension, socioeconomic status, and obesity as possible factors associated with a higher risk of NASH among Iranians.

## Introduction

 Liver cirrhosis is a life-threatening condition accounting for the annual death of more than one million people worldwide, which has increased in the last decades.^1^ Various conditions can cause liver cirrhosis, including viral infections, such as hepatitis B and C, autoimmune diseases, autoimmune hepatitis, liver storage diseases, and fatty liver disease, such as nonalcoholic fatty liver disease (NAFLD).^2^

 With a global prevalence of 24%, NAFLD is one of the most common and preventable causes of liver cirrhosis worldwide.^3,4^ However, with an estimated prevalence of 32%, the prevalence of NAFLD is higher in the Middle East than in other regions.^4^ Histologically, NAFLD is classified as nonalcoholic fatty liver (NAFL) or nonalcoholic steatohepatitis (NASH).^5,6^ NASH is characterized by ≥ 5% hepatic steatosis and inflammation accompanied by hepatocyte damage, with or without fibrosis.^5^ NASH is a severe form of liver injury that can be a risk factor for progressive fibrosis, cirrhosis, and end-stage liver disease.^7^ With an estimated prevalence of 5.85%, the North Africa and Middle East region has one of the highest prevalences of NASH in the world.^8^ In a population-based study in Iran, the prevalence of NASH was estimated to be 2.9% among Iranian people, and NASH prevalence varied considerably across different provinces of Iran.^9^ Furthermore, this study found that being male, overweight or obese, and living in urban areas were each independently associated with higher risk of NASH among Iranian individuals.^9^

 NASH is more prevalent among the elderly, patients with morbid obesity, and people with diabetes. High cholesterol, high triglycerides, and metabolic syndrome are also among the risk factors of NASH.^4,10^ An autopsy study on 351 nonalcoholic patients estimated an 18.5% prevalence of NASH among obese patients.^11^ More recent studies assessing the prevalence of NASH in healthy individuals who were liver donors found that the prevalence of NASH varies from 1.1% to 14%.^12-14^

 It is crucial to understand the prevalence of NASH and its associated factors in the populations, as this information assists policymakers in the health field in passing legislation to reduce health system costs and the burden of severe liver disease. However, studies on the prevalence of NASH and the factors associated with its high prevalence among Iranian people are limited. Therefore, this study was conducted with two main objectives, which are to determine the prevalence of probable NASH and to identify the factors associated with a higher risk of probable NASH among a sample of Iranian adults using data from the Pars Cohort Study (PCS).

## Materials and Methods

###  Design

 This cross-sectional study was conducted based on PCS. The ethics committee of Tehran University of Medical Sciences approved the study protocol (ethics code: IR.TUMS.MEDICINE.REC.1400.566).

###  Participants and Setting

 PCS is a prospective research on the prevalence and risk factors of non-communicable diseases in the southern Iranian region of Fars. The detailed methodological aspects of the study are reported elsewhere, and we explain them briefly here.^15^ Beginning in the fall of 2012 in Valashahr and the surrounding villages in southern Fars province, a team of general practitioners, nurses, nutritionists, lab technicians, data entry staff, and receptionists operated the project at the PCS center. Additionally, native *Behvarz* were trained to participate in conducting the study and in follow-ups. Valashahr includes 40 000 inhabitants, mostly of two ethnicities: Persian and Azari. All 40-75-year-old residents (9721 individuals) in the Valashahr area were invited to participate in the study, with 9264 subjects participating in the study (response rate = 95%). Individuals whose data required for the current study were missing were excluded from the current study. Also, those with an active hepatitis C virus (HCV) infection (confirmed by measuring the antibody against it), hepatitis B virus (HBV) infection (confirmed by measuring hepatitis B virus surface antigen (HBs Ag) levels), or history of alcohol consumption were excluded from the study. Informed consent was obtained from the participants before their participation in the study.

###  Variables

 In our cross-sectional study, we employed baseline PCS data. A standardized questionnaire, physical examination, and blood sample were used in PCS to collect data. Expert computer operators, trained to use the software designed for PCS, carried out the data entry process.

 Questionnaires were completed through interviews by local physicians and well-trained nurses were used to collect data on basic and demographic variables, including age, gender, current place of residence, marital status, ethnicity, and educational level. There were also questions regarding the past medical history, history of gastrointestinal symptoms, source of drinking water, history of smoking, and history of opioid use.

 Participants were asked two questions about their smoking history. First, they were asked if they had ever smoked at least once a week for six months during their lifetime. Second, they were asked if they currently smoked. Those who answered yes to the second question were classified as current smokers, those who answered no to both questions were classified as never smokers, and those who answered yes to the first question but no to the second were classified as past smokers. Regarding opioid use, participants were asked if they had ever used opioids at least once a week for six months during their lifetime

 There were questions in the questionnaires regarding the ownership of a house, car, motorcycle, color television, black and white television, bathroom in the house, vacuum cleaner, washing machine, refrigerator, freezer, computer, and microwave. We also asked them about the room per capita in their family. We used this information to perform a principal component analysis (PCA) and calculate the wealth index to evaluate participants’ socioeconomic status. They were afterward categorized into five quantiles based on their wealth index values.

 Participants’ past medical history was assessed by asking if they had ever been diagnosed with any of the following conditions: heart disease, stroke, hypertension, diabetes, obstructive lung disease, renal failure, cancer, anxiety, depression, or if they had ever received a blood transfusion. Additionally, they were asked if they had ever experienced jaundice or any of the following gastrointestinal symptoms in the past year: heartburn, regurgitation, diarrhea, or bloody diarrhea.

 Qualified medical providers performed medical examinations. Blood pressure was measured in a seated position following a 5-minute rest. Two measurements were taken from each arm with a two-minute delay between them. The mean of the two measurements was considered as the measured blood pressure for that arm. Also, we considered the systolic and diastolic blood pressures of the arm, which were higher than the other as participants’ systolic and diastolic blood pressure. Diastolic blood pressure of 90 or higher and systolic blood pressure of 140 or higher were considered elevated blood pressure. In the regression analysis, elevated blood pressure or a history of diagnosed hypertension were considered as having hypertension.

 We measured participants’ weight, height, and waist circumference (WC). Body mass index (BMI) was calculated by dividing weight in kilograms by height in meters squared. BMI was categorized as underweight (less than 18.5), normal (18.5‒24.9), overweight (25‒29.9), and obese (30 or more). WC of greater than 102 centimeters or higher for males and 88 centimeters or higher for females were considered elevated.

 All participants were instructed to fast for 12 hours prior to entering the center. The 15-mL blood sample was collected after participants’ registrations in the study. Using an ethylenediamin tetra-acetic acid (EDTA) tube, blood was drawn. Blood biochemical parameters were determined using an Autoanalyzer, model BT1500, and Pars Azmoon Company (Iran) kits. White blood cell (WBC) count, hemoglobin, platelets (PLT) count, fasting blood sugar (FBS), triglyceride (TG), high-density lipoprotein (HDL), low-density lipoprotein (LDL), total cholesterol (TC), asparagine aminotransferase (AST), alanine aminotransferase (ALT), alkaline phosphatase (ALP), and gamma glutamyl transpeptidase (GGT) were measured. None of the participants had active HBV or HCV infection, as confirmed by the laboratory tests. Therefore, we defined probable NASH as elevated ALT levels higher than 40 (U/L).^9^

###  Statistical Analysis

 We calculated the mean and standard deviation (SD) for continuous variables. Kolmogorov-Smirnov test was used to determine if the continuous variables were distributed normally, and as all of them had non-normal distributions (*P* < 0.05), we used the Mann-Whitney test to compare the continuous variables in individuals with and without NASH. Frequency and percentage were calculated for categorical variables. We used Chi-squared or Fisher exact tests to compare the categorical variables in individuals with and without NASH. Multiple stepwise backward binary logistic regressions were used to determine the sociodemographic characteristics (gender, age, marital status, place of residence, ethnicity, educational level, wealth index, source of driniking water, history of smoking, and history of opioid use) underlying health conditions (heart disease, stroke, hypertension, diabetes, obstructive lung disease, renal failure, cancer, anxiety, and depression), and anthropometric measures (BMI and WC) associated with probable NASH in participants. We considered the *P* value < 0.05 statistically significant. All analyses were performed using SPSS version 26.

## Results

 Overall, 8734 patients, including 3917 men (44.8%) and 4817 women (55.2%), were enrolled in this study. Among the participants, 605 (6.9%) had NASH. The prevalence of NASH was slightly higher in men compared to women (9.5 vs. 4.8), which shows a statistically significant difference (*P* < 0.001). The average age of participants was 52.62 years (SD = 9.68), and patients with NASH were significantly younger than those without NASH (mean age of 49.32 vs. 52.87 years, *P* < 0.001). Our findings revealed that the prevalence of NASH was higher among married participants, individuals with higher education levels, those in higher wealth index quantiles, and those with a history of smoking (*P* < 0.05). The association between the sociodemographic characteristics of participants and NASH is presented in Table 1.

**Table 1:** Basic and Sociodemographic Characteristics of Participants with and without NASH

**Variable**		**Number (% of Total)/Mean (SD)**	**Without NASH**	**With NASH**	***P *** **Value**
Gender	Male	3917 (44.8%)	3543 (90.5%)	374 (9.5%)	< 0.001
	Female	4817 (55.2%)	4586 (95.2%)	231 (4.8%)	
Age (y)		52.62 (9.68)	52.87 (9.75)	49.32 (7.99)	< 0.001
Marital status	Single	261 (3%)	246 (94.3%)	15 (5.7%)	0.033
	Married	7750 (88.7%)	7194 (92.8%)	556 (7.2%)	
	Divorced or widow	723 (8.3%)	689 (95.3%)	34 (4.7%)	
Ethnicity	Persian	4891 (56%)	4541 (92.8%)	350 (7.2%)	0.351
	Other	3843 (44%)	3588 (93.4%)	255 (6.6%)	
Plece of residence	Urban	1181 (13.5%)	1078 (91.3%)	103 (8.7%)	0.011
	Rural	7552 (86.5%)	7050 (93.4%)	502 (6.6%)	
Educational level	Illiterate	4342 (49.7%)	4127 (95%)	215 (5%)	< 0.001
	1-5 years of education	2583 (29.6%)	2410 (93.3%)	173 (6.7%)	
	6-8 years of education	887 (10.2%)	792 (89.3%)	95 (10.7%)	
	9 or more years of education	922 (10.6%)	800 (86.8%)	122 (13.2%)	
Wealth index	Poorest	1758 (20.2%)	1664 (94.7%)	94 (5.3%)	< 0.001
	Second poor	1765 (20.2%)	1691 (95.8%)	74 (4.2%)	
	Middle	1715 (19.7%)	1602 (93.4%)	113 (6.6%)	
	Second Rich	1769 (20.3%)	1620 (91.6%)	149 (8.4%)	
	Richest	1716 (19.7%)	1541 (89.8%)	175 (10.2%)	
Source of drinking water	Pipe	8023 (91.9%)	7452 (92.9%)	571 (7.1%)	0.017
	Other	711 (8.1%)	677 (95.2%)	34 (4.8%)	
Smoking	Never	7049 (80.7%)	6586 (93.4%)	463 (6.6%)	0.006
	In the past	575 (6.6%)	518 (90.1%)	57 (9.9%)	
	Current	1110 (12.7%)	1025 (92.3%)	85 (7.7%)	
History of opioid use	No	8103 (92.8%)	7543 (93.1%)	560 (6.9%)	0.807
	Yes	631 (7.2%)	586 (92.9%)	45 (7.1%)	

NASH, nonalcoholic steatohepatitis; SD, standard deviation.

 The association between the past medical history of participants and NASH is shown in Table 2. The prevalence of NASH was higher among individuals with a history of diabetes (*P*= 0.017). However, there was no other significant association between the history of other diseases and NASH among the participants (*P* > 0.05).

**Table 2:** Past Medical History of Participants with and without NASH

**Variable**		**Number (% of Total)**	**Without NASH**	**With NASH**	***P *** **Value**
History of heart disease	No	7826 (89.6%)	7298 (93.3%)	528 (6.7%)	0.053
	Yes	908 (10.4%)	831 (91.5%)	77 (8.5%)	
History of stroke	No	8576 (98.2%)	7977 (93%)	599 (7%)	0.152
	Yes	158 (1.8%)	152 (96.2%)	6 (3.8%)	
History of hypertension	No	7301 (83.6%)	6799 (93.1%)	502 (6.9%)	0.649
	Yes	1433 (16.4%)	1330 (92.8%)	103 (7.2%)	
History of diabetes	No	7909 (90.6%)	7378 (93.3%)	531 (6.7%)	0.017
	Yes	825 (9.4%)	751 (91%)	74 (9%)	
History of obstructive lung disease	No	8398 (96.2%)	7818 (93.1%)	580 (6.9%)	0.662
	Yes	336 (3.8%)	311 (92.6%)	25 (7.4%)	
History of renal failure	No	8639 (98.9%)	8043 (93.1%)	596 (6.9%)	0.308
	Yes	95 (1.1%)	86 (90.5%)	9 (9.5%)	
History of cancer	No	8683 (99.4%)	8081 (93.1%)	602 (6.9%)	0.581
	Yes	50 (0.6%)	48 (96%)	2 (4%)	
History of anxiety	No	6128 (70.2%)	5694 (92.9%)	434 (7.1%)	0.407
	Yes	2606 (29.8%)	2435 (93.4%)	171 (6.6%)	
History of depression	No	7039 (80.6%)	6539 (92.9%)	500 (7.1%)	0.201
	Yes	1695 (19.4%)	1590 (93.8%)	105 (6.2%)	
History of blood transfusion	No	7988 (91.5%)	7435 (93.1%)	553 (6.9%)	0.94
	Yes	746 (8.5%)	694 (93%)	52 (7%)	

NASH, nonalcoholic steatohepatitis.

 The association between gastrointestinal symptoms and NASH is shown in Table 3. The prevalence of NASH was higher among individuals who had experienced heartburn or regurgitation in the past year prior to the study (*P* < 0.05). However, there was no other significant association between gastrointestinal symptoms and NASH among the participants (*P* > 0.05).

**Table 3:** Gastrointestinal symptoms in participants with and without NASH

**Variable**		**Number (% of Total)**	**Without NASH**	**With NASH**	***P *** **Value**
History of jaundice	No	8547 (97.9%)	7956 (93.1%)	591 (6.9%)	0.77
	Yes	187 (2.1%)	173 (92.5%)	14 (7.5%)	
Heartburn in the past year	No	5955 (68.2%)	5565 (93.5%)	390 (6.5%)	0.046
	Yes	2779 (31.8%)	2564 (92.3%)	215 (7.7%)	
Regurgitation in the past year	No	4255 (48.7%)	3985 (93.7%)	270 (6.3%)	0.039
	Yes	4477 (51.3%)	4142 (92.5%)	335 (7.5%)	
Diarrhea in the past year	No	8637 (98.9%)	8039 (93.1%)	598 (6.9%)	0.837
	Yes	94 (1.1%)	87 (92.6%)	7 (7.4%)	
Bloody diarrhea in the past year	No	8681 (99.5%)	8079 (93.1%)	602 (6.9%)	0.365
	Yes	43 (0.5%)	42 (97.7%)	1 (2.3%)	

NASH, nonalcoholic steatohepatitis.

 Laboratory tests and obesity indices associations with NASH are shown in Table 4. Higher BMIs were associated with a higher risk of having NASH (*P* < 0.001). However, there was no association between WC and NASH (*P* = 0.05). FBS, TG, cholesterol, LDL, ALT, AST, ALP, GGT, WBC, and hemoglobin levels were all significantly higher among individuals who had NASH (P < 0.001).

**Table 4:** Results of Physical Examinations and Laboratory Tests in Participants with and without NASH

**Variable**		**Number (% of total)/ mean (SD)**	**Without NASH**	**With NASH**	***P*** **-Value**
Elevated blood pressure	No	6938 (79.4%)	6474 (93.3%)	464 (6.7%)	0.085
	Yes	1796 (20.6%)	1655 (92.1%)	141 (7.9%)	
Body mass index (kg/m^2^)	Underweight	368 (4.2%)	361 (98.1%)	7 (1.9%)	< 0.001
	Normal	3514 (40.3%)	3360 (95.6%)	154 (4.4%)	
	Overweight	3254 (37.3%)	2961 (91%)	293 (9%)	
	Obese	1592 (18.2%)	1441 (90.5%)	151 (9.3%)	
Waist circumference	Normal	4983 (57.1%)	4661 (93.5%)	322 (6.5%)	0.050
	Increased	3751 (42.9%)	3468 (92.5%)	283 (7.5%)	
Fasting blood sugar (mg/dL)		105.69 (37.08)	105.25 (36.61)	111.56 (42.59)	< 0.001
Triglyceride (mg/dL)		154.3 (101.67)	150.77 (97.34)	201.76 (139.75)	< 0.001
Cholesterol (mg/dL)		195.72 (41.72)	194.89 (41.46)	206.82 (43.58)	< 0.001
HDL (mg/dL)		57.89 (13.08)	58.09 (13.02)	55.17 (13.56)	< 0.001
LDL (mg/dL)		107.39 (33.5)	107.06 (33.16)	111.86 (37.61)	0.001
AST (U/L)		18.86 (7.72)	17.73 (5.27)	34.02 (15.48)	< 0.001
ALT (U/L)		20.97 (13.84)	18.25 (7.5)	57.45 (24.12)	< 0.001
Alkaline phosphatase (U/L)		266.93 (83.53)	265 (80.39)	290.37 (115.47)	< 0.001
Gamma-glutamyl Transferase (U/L)		26.55 (24.76)	24.52 (20.42)	53.79 (50.58)	< 0.001
WBC (*10^9^/L)		6.47 (1.95)	6.44 (1.91)	6.89 (2.44)	< 0.001
Hemoglobin (g/dL)		13.73 (1.74)	13.7 (1.73)	14.21 (1.75)	< 0.001
Platelet (*10^3^/microL)		243.24 (70.52)	243.4 (70.53)	241.14 (70.36)	0.782

ALT, alanine aminotransferase; AST, aspartate aminotransferase; HDL, high-density lipoprotein; LDL, low-density lipoprotein; NASH, nonalcoholic steatohepatitis; SD, standard deviation; WBC, white blood cell.

 The results of logistic regressions determining the factors associated with NASH are presented in Table 5. Male gender, younger age, history of heart disease, history of diabetes, hypertension, being overweight or obese, being in the richest or second richest wealth index quantiles, and increased WC were independently associated with a higher risk of having NASH (*P* < 0.05).

**Table 5:** Results of Multiple Stepwise Backward Binary Logistic Regressions Determining the Sociodemographic Characteristics and Underlying Health Conditions Associated With Probable NASH in Participants

**Variable**	**OR**	**95% CI**	***P *** **Value**
Age	0.951	0.941‒0.962	< 0.001
Female gender	0.31	0.249‒0.386	< 0.001
History of heart disease	1.499	1.146‒1.962	0.003
History of diabetes	1.523	1.162‒1.995	0.002
Hypertension	1.241	1.023‒1.506	0.029
Being overweight or obese	2.192	1.755‒2.737	< 0.001
Being in the richest or second richest wealth index groups	1.315	1.107‒1.562	0.002
Increased waist circumference	1.409	1.107‒1.793	0.005

95% CI, 95% confidence interval; OR, odds ratio.

## Discussion

 The causes of NASH have not been clarified yet. Consequently, treatment and diagnosis present considerable challenges. In this study, we analyzed different variables that may contribute to the global prevalence of NASH and found that age, gender, wealth index, and clinical conditions such as diabetes, hypertension, and obesity are associated with NASH.

 NASH can affect individuals of various ages; however, in our population study, as a novel finding, age and NASH prevalence exhibit an inverse relationship (OR = 0.951, 95% CI 0.941‒0.961) in contrast to earlier studies indicating aging as a risk factor for NASH.^16^ A study by Ghassemi et al revealed alterations in the Iranian diet and increased fat and sugar consumption, particularly among the younger population.^17^ Dietary changes and westernization in Iran, which has started in the past decades,^17,18^ might be a reason for the higher prevalence of NASH among younger Iranian adults as older adults might have maintained their healthy and traditional lifestyle and diet. Further studies on other Iranian populations are needed to evaluate the possible association of dietery and lifestyle changes in younger generations and the development of NASH.

 The prevalence of NASH was higher among males than females in our study, which is in line with previous studies.^19^ Differences in the prevalence of obesity and lifestyle-related diseases explain gender differences in developing NASH.^20^ After menopause, the prevalence of NASH increases among females. A greater risk of NASH in men and postmenopausal women compared to premenopausal women, as well as experimental studies on the effects of estrogen on the liver, suggest that estrogen might have a protective role in the development of NASH.^21-23^

 We found that NASH is more common among people with better socioeconomic status. The consumption of high-calorie foods, such as fast food, and insufficient physical activity in wealthy communities can contribute to this disparity between poor and wealthy populations regarding NASH prevalence.^24^ However, the research by Abougergi et al revealed that this relation is generally independent of well-known risk factors like obesity and diabetes, and the correlation between NASH prevalence and living in dense counties is significantly larger than that between wealth and food security.^25^

 As indicated in our regression analysis, higher BMIs and WCs are both associated with a higher risk of having NASH. Previous studies have also indicated that obesity is a risk factor for NAFLD.^26^ As reported, liver biopsies during bariatric surgeries show a high prevalence of NAFLD and NASH in severe and morbidly obese individuals.^27-29^ Recently, NASH has been noted as one of the most common consequences of metabolic syndrome associated with obesity.^30,31^ Fatty liver is also associated with increased visceral fat accumulation, which can be measured by WC.^32^ Previous studies have estimated the prevalence of obesity to be 17.4% among Iranians, which has increased in the past years.^33^ Similarly, about 18% of our participants were obese as measured by BMI, and WC was increased in about 43% of them, which is concerning. Therefore, considering the association between the development of NASH and obesity, the prevalence of NASH might consequently increase in Iran in the coming years, which warrants actions by policymakers.

 We found that the prevalence of NASH is higher among individuals with heartburn and regurgitation. In the study by Wijarnpreecha et al, they reported a significantly elevated risk of NAFLD among GERD patients, with a pooled OR of 2.07 (95% confidence interval: 1.54–2.78).^34^ It has been shown that increased abdominal pressure due to visceral fat deposition contributes to esophageal regurgitation and the development of GERD.^35^ Unhealthy eating habits, such as consuming large meals before sleep, can contribute to both GERD and obesity. Triglyceride may impact the tone of the lower esophageal sphincter and may be the common underlying factor linking NAFLD and GERD.^36,37^ Furthermore, visceral adipose tissue in humans is known to produce many proinflammatory cytokines, and an elevated amount of these cytokines is connected with a decreased esophageal sphincter tone, which may predispose individuals to GERD.^38,39^

 This study was one of the few large-scale studies on the prevalence of NASH and the associated factors among Iranians, which provides insight into the current status of diseases and the associated factors among Iranians. However, there are several limitations to this article worth mentioning. First, we used laboratory criteria to diagnose NASH, and studies using more definite criteria, such as histological assessments, are needed in the future. This limitation could lead to an overestimation of NASH prevalence in the current study. Second, our study was cross-sectional, which can not demonstrate the causal relationship between variables. Therefore, future longitudinal studies are indicated to determine the risk factors of NASH among Iranians. Moreover, part of the PCS data was derived from self-reported questionnaires, which may lead to reporting bias and recall bias. Finally, we did not assess physical activity and nutrition status in the current study, which may be counted as another limitation.

## Conclusion

 In this study, we determined the prevalence of NASH and found gender, age, history of heart disease, history of diabetes, hypertension, socioeconomic status, and obesity as possible factors associated with a higher risk of NASH among Iranians. Considering the increasing trend of obesity in Iran and the association of obesity with NASH, preventive strategies are needed to avoid an increase in the prevalence of NASH among Iranians. Also, further studies are needed to evaluate the association between age and NASH among Iranians, considering the disparity between our findings and previous studies worldwide and the possible roles of diet and lifestyle in the development of NASH among younger Iranians.
